# Aldosterone signaling defect in young infants: single-center report and review

**DOI:** 10.1186/s12902-021-00811-9

**Published:** 2021-07-09

**Authors:** Melati Wijaya, Huamei Ma, Jun Zhang, Minlian Du, Yanhong Li, Qiuli Chen, Song Guo

**Affiliations:** Department of Pediatrics, The First affiliated Hospital, Sun Yat-Sen University, No. 58, Zhongshan II Rd, Guangzhou, 510080 P. R. China

**Keywords:** Salt wasting, Infant, Primary adrenal insufficiency, Adrenal disease, Aldosterone synthase deficiency, Pseudohypoaldosteronism

## Abstract

**Background:**

Aldosterone (Ald) is a crucial factor in maintaining electrolyte and water homeostasis. Defect in either its synthesis or function causes salt wasting (SW) manifestation. This disease group is rare, while most reported cases are sporadic. This study aimed to obtain an overview of the etiology and clinical picture of patients with the above condition and report our rare cases.

**Methods:**

A combination of retrospective review and case studies was conducted at the Pediatric Endocrine unit of The First Affiliated Hospital Sun Yat Sen University from September 1989 to June 2020.

**Results:**

A total of 187 patients with SW were enrolled, of which 90.4% (*n* = 169) were diagnosed with congenital adrenal hyperplasia (CAH). SW type 21-hydroxylase deficiency accounted for 98.8% (*n* = 167) of CAH diagnosis, while 1.2% (*n* = 2) was of lipoid CAH. Non-CAH comprised 9.6% (*n* = 18) of the total patients whose etiologies included SF-1 gene mutation (*n =* 1), X-linked adrenal hypoplasia congenita (*n* = 9), aldosterone synthase deficiency (ASD, *n* = 4), and pseudo-hypoaldosteronism type 1 (PHA1, *n* = 1). Etiologies were not identified in three patients. All of patients with ASD and PHA1 exhibited SW syndrome in their early neonatal period. DNA sequencing showed mutations of *CYP11B2* for P1-P4 and *NR3C2* for P5. P1 and P2 were sibling brothers affected by compound heterozygous mutations of c.1121G > A (p.R374Q) and c.1486delC p.(L496fs); likewise, P4 was identified with compound heterozygous mutations of c.1200 + 1G > A and c.240–1 G > T; meanwhile P3 demonstrated c.1303G > A p.(G435S) homozygous mutation in *CYP11B2* gene. Lastly, P5 showed c.1768 C > T p.(R590*) heterozygous mutation in the *NR3C2* gene.

**Conclusion:**

Etiology of infant with aldosterone defect was mostly congenital. Renal and adrenal imaging are recommended to exclude renal causes. If clinical picture is suggestive, normal plasma Ald in early infancy cannot rule out aldosterone insufficiency.

## Background

Water and electrolyte are crucial components in early postnatal life. 70% of a full-term infant’s body is comprised of water while water content decreases to 60% in an adult body. On the other hand, sodium is the major electrolyte component in extracellular fluid (ECF) and plays an important role in maintaining intracellular and extracellular stability. A positive net balance of sodium and water is needed for an infant’s stable growth [1].

Aldosterone (Ald), which is synthesized by the adrenal cortex, plays an important role in controlling sodium (Na), potassium (K), chloride (Cl), hydrogen ion, and water homeostasis in humans. Ald regulates fluid and electrolyte balance by modulating renal tubular reabsorption of Na, Cl, water, and excretion of potassium. Ald signaling defect, including impairment of Ald synthesis or function, causes salt wasting (SW) manifestation typically characterized by hyponatremia, metabolic acidosis, hypovolemia with or without hyperkalemia [2, 3].

Defects in Ald function (secondary hypoaldosteronism or pseudohypoaldosteronism, PHA) are related to functional abnormalities of the mineralocorticoid receptor (MR) or abnormalities in ion transport channel that responds to Ald in the distal renal tubule [3]. PHA also occurs due to pyelonephritis, tubulointerstitial nephritis, obstructive uropathy in infants and children which can be seen in 3% of those with urinary tract infection (secondary PHA) [4, 5]. Clinical symptoms and biological parameters return to normal after resolution of the infection.

Reduced synthesis of Ald (primary hypoaldosteronism) may be the result of genetic mutation leading to disability in producing enzymes or caused by acquired destruction of the adrenal cortex. Hypoaldosteronism due to genetic disorder is commonly seen in primary adrenal insufficiency (PAI). Its most common etiology is congenital adrenal hyperplasia (CAH) due to 21-hydroxylase (21-OH) deficiency that accounts for 75–80% of total diagnoses [6, 7]. In addition, other rare etiologies such as familial glucocorticoid deficiency (FGD), X-linked adrenal hypoplasia congenita (X-AHC), aldosterone synthase deficiency (ASD) also causes SW. Some etiologies, such as ASD, have distinct characteristics, wherein the affected children present with SW but the glucocorticoid function is normal.

SW in newborns and infants is relatively common and requires emergency therapy. Numerous etiologies both from renal and adrenal disorders may present with similar manifestations which lead to confusion in establishing a specific diagnosis. This study was aimed to summarize the etiologies of Ald defect in infants under 1 year old with SW manifestation. We also described four cases of ASD, as well as one case PHA type 1 (PHA1) in addition to literature study.

## Methods

### Data collection

A retrospective review was conducted at The First Affiliated Hospital, Sun Yat Sen University pediatric endocrinology unit between September 1989 and June 2020. The diagnostic categories were as follows: 21-OH deficiency, Lipoid CAH (LCAH), SF-1 gene mutation, X-AHC, ASD, PHA1, PAI with SW manifestation.

The selection criteria were as follows: (1) clinical signs and symptoms suggesting endocrine origin, such as recurrent vomiting, hyperpigmentation; (2) hyponatremia (serum Na < 130 mEq/L) with or without hyperkalemia during the first year of life; (3) a positive gene test indicating one of the etiologies of Ald synthesis or function defect by sequencing the appropriate candidate gene or by using multiplex ligation-dependent probe amplification (MLPA) or whole exome sequencing (WES) technology [1, 2, 8]. Patients with PAI without SW manifestation; hyponatremia due to gastrointestinal disease, renal disease, central nervous disease, feeding difficulties with/without inadequate intake were excluded. PAI was diagnosed according to endocrine society practical guideline for PAI [8]. Diagnosis of 21-OH deficiency SW type was based on CAH clinical practical guideline [9]. Diagnosis of other rare diseases was based on clinical manifestation, biochemical test, and gene testing result. The following data were collected from medical records: age at presentation, sex, clinical manifestation, biochemical and adrenal hormonal features at initial presentation, adrenal imaging, and result of genetic mutation analysis.

Description of clinical manifestation, biochemical characteristics, and outcome of four patients from three different Chinese families with ASD who demonstrated positive results of *CYP11B2* analysis and one case of PHA1 due to *NR3C2* mutation were also studied. Written informed consent for genetic analysis was obtained from their parents according to institutional ethical guidelines.

### *CYP11B2* and *NR3C2* genetic study

The genomic DNA of the patients and their parents were isolated from peripheral blood leukocytes using QIAamp DNA blood mini kits. *CYP11B2* (OMIM 124080) exons 1–9 and their respective exon-intron boundaries were amplified by PCR and analyzed by direct sequencing. The resulting sequencing data was compared with the reference sequence (NM_000498.3) and mutations were checked in the Human Gene Database (HGMD).

*NR3C2* (OMIM 177735) exons and intron regions were amplified by PCR and analyzed by direct sequencing. The resulting sequencing data was compared with the reference sequence (NM_000901.4).

## Results

### Demography

A total of 187 patients (124 males, 63 females) were enrolled in this study. The median age at presentation was 0.08 (range 0.02 to 0.91) years. 87 patients were diagnosed based only on their clinical presentation, 97 patients were genetically confirmed, while 3 patients did genetic testing without positive results.

### Etiology

The final diagnosis for 169 patients (90.4%) was that of CAH, out of which 98.8% (*n* = 167) was based on clinical findings with/without genetic testing diagnosis as 21-OH deficiency SW type. Meanwhile, two patients (1.2% of CAH) were genetically confirmed with LCAH (Table 1).

**Table 1:** Etiology of aldosterone defect in infants

Etiology	Male *n* = 124	Female *n* = 63	Total *n* = 187	% of total
CAH	111	58	169	90.4%
21-OH-CAH SW type	110	57	167	
Lipoid CAH	1	1	2	
Non-CAH	13	5	18	9.6%
SF-1 gene mutation	0	1	1	
X-linked AHC	9	0	9	
ASD	2	2	4	
PHA1	1	0	1	
Unknown etiology	1	2	3	

*CAH* congenital adrenal hyperplasia, *21-OH-CAH SW type* 21-hydroxylase deficiency CAH salt wasting type, *SF-1* steroidogenic factor 1, *AHC* adrenal hypoplasia congenita, *ASD* aldosterone synthase deficiency, *PHA1* pseudohypoaldosteronism type 1

Eighteen patients (9.6%) who had done genetic testing were affected by non-CAH form of endocrine origin (Table 2). One patient carried *NR5A1* (SF-1) mutation. In addition, nine patients who demonstrated *NROB1* gene mutation were diagnosed as X-AHC. Whereas four patients from three unrelated families were confirmed as ASD, one patient was confirmed as renal PHA1. In three patients (one male and two females) with clinical and hormonal diagnosis of PAI, the final genetic etiology was not identified by WES.

**Table 2:** Characteristics of infants with aldosterone defect non 21-OH CAH adrenal etiology

No	Sex	Age at onset	Clinical presentation	Na mmol/L (135 ~ 145)	K mmol/L (3.5 ~ 5.0)	Diagnosis	Gene	Mutation
1	M	1 m	Poor feeding	130	6.3	X-AHC	*NROB1*	c.1168 + 1_1168 + 2dupGT
2	M	10 d	Salt wasting crisis, hyperpigmentation	120	5.8	X-AHC	*NROB1*	c.791_793delAGA,p.(K264del)
3	M	15 d	Recurrent vomiting, hyperpigmentation	130	5.0	X-AHC	*NROB1*	c.460A > T p.(K154*)
4	M	10 d	Recurrent vomiting	130	7.58	X-AHC	*NROB1*	c.332_333delCT p.(S111*)
5	M	1 m	Failure to thrive, recurrent vomiting, diarrhea	126	6.3	X-AHC	*NROB1*	c.1231_1234delCTCA p.(K411fs)
6	M	5 d	Diarrhea, hyperpigmentation	128	6.9	X-AHC	*NROB1*	c.332_333delCT p.(S111*)
7	M	1 m	Poor feeding, failure to thrive, hyperpigmentation	115	7.49	X-AHC	*NROB1*	E1-E2 del
8	M	5 d	Vomiting, diarrhea, dehydration, hyperpigmentation	110	3.0	X-AHC	*NROB1*	c.585_595del11 p.(Y97fs)
9	M	1 m	Poor feeding	134	6.57	X-AHC	*NROB1*	c.838delC p.(L280fs)
10	F (46,XY)	1 m	Failure to thrive, hyperpigmentation	132	7.2	SF-1 mutation	*NR5A1*	c.616-758del
11	M	6 m	Vomiting, hyperpigmentation	129	6.2	LCAH	*STAR*	c.65C3C > T, p.(A218V) homozygous
12	F	6 m	Adrenal crisis	114	5.6	LCAH	*STAR*	Heterozygous c.367G > A, p.(E123K) and c.465 + 2 T

*M* male, *F* female, *d* days, *m* months, *X-AHC* X-linked adrenal hypoplasia congenita, *LCAH* lipoid congenital adrenal hyperplasia

### Clinical presentation

The symptoms of Ald defect in infants were vomiting (70.8%), failure to thrive (54.6%), poor feeding (21.6%), diarrhea (17.3%), dehydration (11.4%), and seizure (4.3%). Nine patients had histories of neonatal death in his or her sibling who presented with similar manifestation.

### Clinical case of aldosterone synthase deficiency

#### Patient 1 (P1)

This was a 2-month-old male patient who was born at term with a birth weight of 3450 g. He was the second child of non-consanguineous Chinese parents. Since 2 weeks old he was admitted to local hospital due to recurrent vomiting, diarrhea, poor feeding, dehydration, hyponatremia, and hyperkalemia. Prompt therapy with sodium chloride (NaCl) and 5% dextrose as well as sodium bicarbonate infusion were initiated, then the patient was transferred to our unit for further investigation.

Physical examination (PE) on admission showed: weight of 4 kg (−1SD), length of 55 cm (0 SD), no dehydration, no hyperpigmentation, and normal external genitalia. The laboratory findings were as follows: Plasma Na 130 mmol/L (Normal Range [NR] 135 ~ 145 mmol/L), K 6.86 mmol/L (NR 3.5 ~ 5.0 mmol/L), Cl 104 mmol/L (NR 96 ~ 106 mmol/L), Adrenocorticotropic hormone (ACTH) 3.37 pmol/L (NR 1.6 ~ 13.9 pmol/L), cortisol < 5μg/dl (NR 5 ~ 20μg/dl), plasma renin activity (PRA) 1019.2 pg/ml (NR adult 4 ~ 24 pg/ml, neonatal renin 2.5–20 times higher than adult [10]), and plasma Ald 117.0 pg/ml (NR infants 1-12 months 50-900 pg/ml [10]). Further analysis showed plasma testosterone (T), progesterone (P), Androstenedione (A4), and 17-hydroxyprogesterone (17-OHP) levels were in the normal range. Urinalysis and CT scan for adrenal and renal were also normal. Patient was treated with fludrocortisone (Fc) 0.1 mg/day along with sodium supplementation 2-3 g/day. Since 6-month-old his mother had stopped sodium supplementation. FC dose had been reduced from 1.5 years old till 1.67 years old and was then fully discontinued by his parents. Currently, he is 5.17 years old with height of 104 cm (− 1.93SD). He is asymptomatic with normal serum electrolyte levels.

#### Patient 2 (P2)

This male patient was the elder brother of P1 and was 28 months older than P1, i.e., 30 months old, with similar manifestation as P1 since 2 weeks old. He was admitted to local hospital with biochemical profile when he was 1.5 months old as follows: hyperkalemia (6.17mmol/L), hyponatremia (130 mmol/L), normal ACTH and cortisol (2.93 pmol/L and 290 nmol/L respectively). His adrenal and renal ultrasound imaging was normal. He was discharged with “unknown origin of salt wasting” diagnosis; received 6 months of oral sodium supplementation with normal growth and serum electrolyte level. After P2 1 had his diagnosis confirmed, we proposed to do physical examination, biochemical and gene tests for him. Currently he is 7.5 years old with height of 137 cm (+ 1.5SD).

#### Patient 3 (P3)

This female patient was born at term with a birth weight of 3200 g. Recurrent vomiting, poor feeding, and dehydration led to admission to local hospital at 1 week old. She was found to have hyperkalemia, hyponatremia, and metabolic acidosis. The patient was initially treated with sodium bicarbonate, sodium polystyrene sulfonate, intravenous stress dose hydrocortisone (HC), among others for presumed adrenal insufficiency. After stabilization, she was discharged from hospital with sodium polystyrene sulfonate to reduce blood potassium level as well as sodium supplementation 4.5 g/day. However, correction of hyponatremia and hyperkalemia remained challenging.

P3 was registered in our unit at 5 months old. PE on admission was as follows: weight of 5.5 kg (<− 3SD), length of 58 cm (<− 3.12SD), no hyperpigmentation, and normal external genitalia. Further biochemical profile revealed low plasma Na level (132 mmol/L), high plasma K level (6.2 mmol/L), normal ACTH level (1.92 pmol/L), cortisol level (3.5μg/dl), high PRA level (1287 pg/ml), and inappropriate normal plasma Ald level (672.11 pg/ml). Meanwhile, plasma T, P, and A4 levels were normal in range. In addition, urinalysis, renal and adrenal ultrasonography were found to be normal. Problems in adrenal hormone production were considered. She was then treated with Fc 0.2 mg/day and sodium supplementation 3-6 g/day. The blood electrolyte was well controlled. Her height at 1 year old was 69.5 cm (− 2.17SD).

Furthermore, P3 was the third child of non-consanguineous Chinese parents. Her elder sister was of normal health, whereas the elder brother had similar manifestations as P3 and died 10 days after birth. From further history investigation, it was discovered that P3’s mother experienced generalized weakness in daily activities.

#### Patient 4 (P4)

This patient appeared to be a normal female infant born at term with a birth weight of 3250 g to non-consanguineous Chinese parents. She was admitted to local hospital due to feeding difficulties, poor response, poor weight gain, and dehydration since 21-day-old. Laboratory tests revealed the following biochemical levels: plasma Na 115 mmol/L, K 6.73 mmol/L, and Cl 85 mmol/L. Prompt therapy with sodium chloride infusion, sodium bicarbonate, calcium gluconate, insulin, HC intravenous, and oral Fc were initiated for presumed CAH. A hormonal workup reported a few days later showed elevated PRA level (27.54 .ng/hr, NR 0.33 ~ 6.4 ng/hr), inappropriate normal plasma Ald level (66.51 pg/ml), and normal levels of ACTH and 17-OHP. Ruling out initial suspicion of CAH, she was discharged with oral Fc 0.1 mg/day and sodium supplementation 1–1.5 g/day. P4’s mother stopped Fc administration when she was 5 months old.

At 15-month-old P4 was admitted to our unit. PE showed: weight of 9.3 kg (−1SD), and length of 76 cm (−1SD). Normal plasma Na level was detected (139 mmol/L) as well as high plasma K level (5.56 mmol/L), high PRA level (443.4 pg/ml), and unappropriated normal plasma Ald level (117.0 pg/ml). She started receiving Fc 0.05 mg/day and sodium 0.7 g/day, which were regularly adjusted depending on PRA level and serum electrolyte. P4 was on routine follow up and on the last visit at 4.5-year-old her weight was 20.5 kg (+1SD) and was 109.8 cm (+1SD) in height with normal serum electrolyte. Oral Fc dose was maintained at 0.05 mg/day since she was 3 years old. Oral sodium supplementation was stopped by age 2.5 years old.

### Clinical case of renal PHA1

Patient 5 (P5) was a 22-day-old male child of non-consanguineous healthy Chinese parents at term with a birth weight of 2950 g. He was admitted to hospital due to poor weight gain, feeding difficulties, and poor responsiveness since 7 days old. His parents did not have any similar symptoms in medical history. Physical examination showed: weight of 3.4 kg (−2SD), length of 53 cm (−1SD), no hyperpigmentation, and normal external genitalia. The laboratory findings were as follows: Plasma Na 131 mmol/L, K 5.88 mmol/L, and Cl 98 mmol/L. Meanwhile, the level of ACTH was normal (7.22 pmol/L) and cortisol level was 7.7μg/dl. Whereas relatively high PRA (6858 ng/L/h) and plasma Ald [1566.79 pg/ml, (NR Newborn: 10-1800 pg/ml, NR infants 1-12 months 50-900 pg/ml)] were detected, plasma T, P, A4, 17-OHP were in normal range. Urinalysis was normal. In addition, adrenal CT scan revealed bilateral hyperplasia. The patient was initially treated with HC 9-10 mg/m^2^.d and FC 0.2 mg/d for presumed CAH. Since 2.25-year-old the dose of FC was gradually reduced and maintained at 0.125 mg/d until 7 years old. His serum electrolyte and serum PRA were normal in range. His height was 110 cm (− 2.8SD) and weight 19.5 kg (− 1.8SD) when he was 7 years old. (Table 3).

**Table 3:** Characteristic of infants with hypoaldosterone and aldosterone resistance

Investigation	Patient 1	Patient 2	Patient 3	Patient 4	Patient 5
Age	14 days	14 days	7 days	21 days	7 days
Manifestation	Recurrent vomiting, diarrhea, poor feeding	Recurrent vomiting, diarrhea, poor feeding	Recurrent vomiting, poor feeding	Poor feeding	Poor feeding, failure to thrive
Plasma Na [NR 135 ~ 145 mmol/L]	130	130	132	115	131
Plasma K [NR 3.5 ~ 5.0 mmol/L]	6.86	6.17	6.2	6.73	5.88
Plasma ACTH [NR 1.6 ~ 13.9 pmol/L]	3.37	2.93	1.92	4.95	7.22
Plasma cortisol [NR 5 ~ 20μg/dl]	< 5	10.5	3.5	n.a	7.7
Plasma PRA [NR^a^]	1019.2	n.a	1287	high	High
Plasma Ald [NR^b^]	117.0	n.a	672.11	66.51	1566.79
Adrenal imaging	normal	normal	n.a	n.a	Hyperplasia
Gene mutation	*CYP11B2* Heterozygous c.1121G > A (p.R374Q), c.1486delC p.(L496fs)	*CYP11B2* Heterozygous c.1121G > A (p.R374Q), c.1486delC p.(L496fs)	*CYP11B2* Homozygous c.1303G > A p.(G435S)	*CYP11B2* Heterozygous c.1200 + 1G > A, c.240-1G > T	*NR3C2* Heterozygous c.1768C > T, p.(R590*)
Diagnosis	ASD	ASD	ASD	ASD	PHA1

*Na* sodium, *K* potassium, *PRA* plasma renin activity, *ACTH* Adrenocorticotropic hormone, *Ald* Aldosterone, *n.a* not available, *NR* normal range, *Age* age at presentation, *ASD* aldosterone synthase deficiency, *PHA1* pseudohypoaldosteronism type 1

^a^Adult: 4-24 pg/ml, Neonatal renin is high, which can be 2.5–20 times higher than that of adults, and these values slowly decline but high values can be seen up until the age of 5 years [10]

^b^Newborn: 10-1800 pg/ml, infant 1-12 month 50-900 pg/ml

### Genetic result

**Fig. 1:**
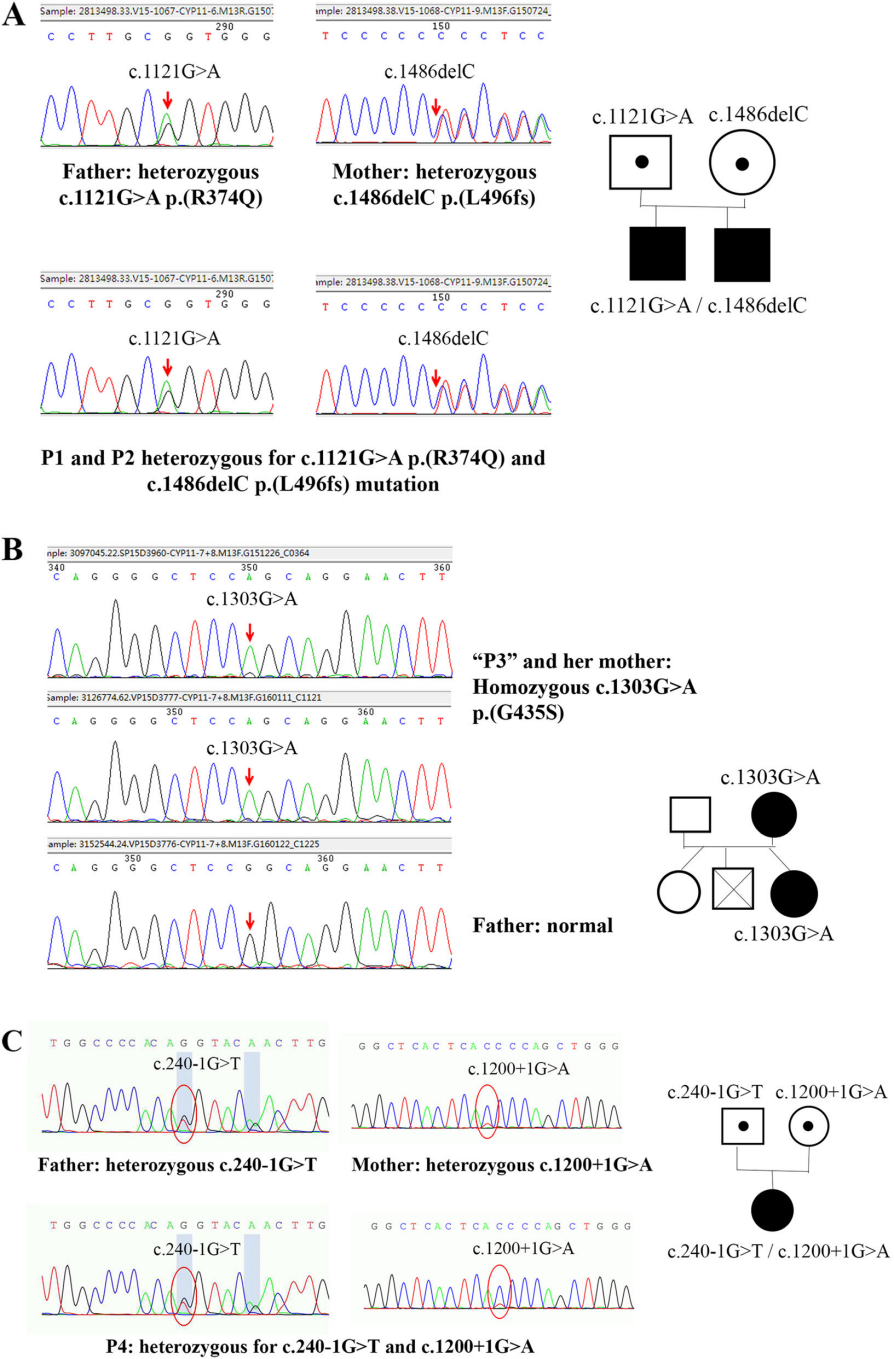
identification of mutation in *CYP11B2* gene. (A) DNA sequence of P1 and P2 family. (B) DNA sequence of P3 family. (C) DNA sequence of P4 family

Four patients were suspected of ASD. P1 and P2 were found to have pathogenic mutations of *CYP11B2* gene: c.1121G > A (p.R374Q) which was inherited from their father and c.1486delC p.(L496fs) that was inherited from their mother. P3 was observed to have homozygous pathogenic mutation of c.1303G > A p.(G435S) in *CYP11B2* gene which she inherited from her mother. Furthermore, P4 was found to have heterozygous pathogenic mutation of c.1200 + 1G > A that was inherited from her mother and c.240–1 G > T which was passed down from her father. (Fig. 1).

**Fig. 2:**
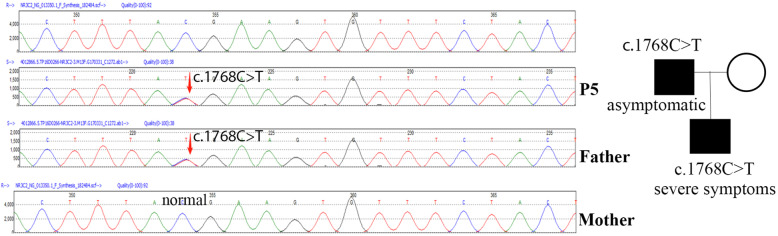
Identification of mutation in *NR3C2* gene. DNA sequence of P5 and his father showed heterozygous mutation of c.1768 C > T p.(R590*), while his mother was normal

P5 was suspected to have PHA1. DNA sequencing revealed a heterozygous mutation of c.1768 C > T p.(R590*) in the *NR3C2* gene which was inherited from his father. (Fig. 2).

## Discussion

There were numerous differential diagnoses for infants with failure to thrive, hyponatremia, and hyperkalemia. In infancy, unless there is clear evidence of gastrointestinal fluid losses or renal dysfunction, these abnormalities usually point to congenital adrenal insufficiency, specifically to CAH with concomitant hypoaldosteronism that accounted for 90.4% of total case. CAH has many variants, of which several of them may only present as virilization both in male and female, sexual infantilism, or hypertension [11]. The other form, such as 21-OH CAH SW (classic) type, presents as SW in infants, and if untreated it can progress to shock and death. Deficiencies of steroidogenic acute regulatory (StAR) protein due to *STAR* gene mutation may also present in SW. This form of CAH is very rare and distinguished clinically by signs of sex steroid deficiency [11, 12].

Several different etiologies were identified in this study. We found one patient with renal form of PHA1 (0.5%) and four patients (2.1%) with ASD. The frequency of ASD in this study was relatively high, considering that the estimated incidence of this defect is probably < 1/1000000/year [3, 13]. The unexpectedly high prevalence in our study suggests frequent underdiagnosis in general population or over-representation in our patient population. The case of X-linked AHC, SF-1 gene mutation, and LCAH were previously described [6].

PHA1 is marked by mineralocorticoid resistance of kidney and/or other mineralocorticoid target issues. The case found in this study was renal-limited PHA1 caused by *NR3C2* mutation which encodes MR. A heterozygous nonsense mutation in exon 3 alters R590 to an immediate stop codon. This haploinsufficiency is sufficient to cause the renal PHA1 [14].

The mutation of R590* has been reported in a pedigree who lived in Spain, in which the clinical presentation was variable, ranging from severe to asymptomatic [14]. As was seen in our case, the patient had severe SW presentation while the father had no history of SW. The mechanism of phenotype variability is still unclear.

We also identified *CYP11B2* gene mutations in this study as follows: one homozygous mutation for c. 1303G > A in P3, a compound heterozygous mutation for c.1121G > A and c.1486delC in P1 as well as P2. The c. 1303G > A was a missense mutation in exon 8 which resulted in substitution of serine for glycine at codon 435. The study of Kuribayashi et al. discovered that the mutation of *G435S* caused drastically reduced in vitro activity of the steroid 18-hydroxylase/oxidase [15]. The c.1121G > A mutation results in a change from arginine to glutamine at codon 374. This mutation is a novel one, known as rs755947763. Based on literature, residue R374 is invariant in the K-helix Glu-X-X-Arg motif, which may be involved in stabilizing the core structure of the enzyme. The point mutation of Glu-X-X-Arg motif causes the change of hydrogen bond which influences substrate recognition and protein stability leading to weakened enzymatic activity of CYP11B2. The c.1486delC is a frameshift mutation and yields a truncated protein, giving rise to the impairment of enzyme activity [16].

We further identified other mutations of the *CYP11B2* gene. Mutation of C.1200 + 1G > A affects a donor splice site in intron 7 of *CYP11B2*, whereas mutation of c.240-1G > T alters an acceptor splice site in intron 1 of *CYP11B2*. Based on literature, donor and acceptor splice site variants typically lead to a loss of protein function [17]. Those two mutations have not been reported.

Most reported cases showed low Ald level in ASD patients, while in this paper we could find that all patients with ASD (P1-P4) showed inappropriate normal of Ald level, similar with the two patients respectively reported by Adriana de Sousa Lages, et al. [18] and Niu li, et al. [17]. The normal ald level in ASD patients may be the result of compensation of Ald secretion due to hyponatremia and hyperkalemia and neonatal hyperactivity of renin-angiotensin-aldosterone system [2]. The condition of high plasma renin level with inappropriate normal range of Ald, hyperkalemia and hyponatremia raising the suspicion of ASD. When the clinical picture is suggestive, normal plasma Ald in early infancy cannot rule out aldosterone insufficiency.

Fludrocortisone (fc) is an essential treatment for patient with mineralocorticoid hormone deficiency. Endocrine society recommends using 0.05–0.2 mg/d divided into 1–2 times/d [19]. Other literature suggested an initial dose of 0.1–0.2 mg/d [2]. The usage of FC dose needs regular adjustment based on clinical condition, plasma sodium, potassium, and renin level; not by body weight. In addition, the patients also need sodium supplementation 1-2 g/d (17–34 mEq/d) during infancy [19]. In most children, the therapy can discontinue when they grow older due to progressive improvement in mineralocorticoid sensitivity and high salt diet. Nonetheless, several patients occasionally showed symptoms.

The termination of mineralocorticoid therapy in ASD patients may lead to postural hypotension and hyperkalemia when triggered by stress due to dehydration or salt intake reduction [20]. Generalized weakness has also been reported in a middle aged man with ASD [21] which was also present in P3’s mother in our study.

Study from Brigitte E, et al. reported that adults carrying NR3C2 mutation tend to have lower HDL and higher waist circumference [22]. This metabolic consequence was thought to be related to activation of the hypothalamo-pituitary-adrenal axis (HPA) system due to haploinsufficiency of MR failing to give negative feedback in the HPA axis that finally induces hypersecretion of cortisol. Further study of this concept is still required. However, the systolic and diastolic blood pressure were normal in spite of high PRA and Ald levels.

Confirmation of specific diagnosis is important because numerous diseases related to Ald defect display similar presentation, albeit requiring different long term clinical management. However, it is necessary for initial treatment of glucocorticoid and mineralocorticoid to start immediately in order to avoid life-threatening complications. In addition to performing comprehensive steroid hormone and genetic testing, we also propose to do renal and adrenal imaging to distinguish renal disorder induced SW.

## Conclusions

This study described the etiologies of Ald defects in our institution’s infants. We also studied four cases of ASD and one case of renal-limited PHA1. The results showed that the most common etiology was of inherited origin. This study had a number of limitations due to its retrospective nature. Nonetheless, this has revealed a knowledge that SW due to Ald defect is not only about CAH but other etiologies are also possible. Constructing multiple differential diagnoses is essential for establishing appropriate care of these infants.

## Data Availability

The datasets used and analyzed during the current study are available from the corresponding author on reasonable request.

## Abbreviations

Ald: Aldosterone
ECF: Extracellular fluid
SW: Salt wasting
PHA: Pseudohypoaldosteronism
PHA1: Pseudohypoaldosteronism type 1
MR: Mineralocorticoid receptor
PAI: Primary Adrenal Insufficiency
CAH: Congenital Adrenal Hyperplasia
21-OH: 21-hydroxylase
FGD: Familial glucocorticoid deficiency
X-AHC: X-linked adrenal hypoplasia congenita
ASD: Aldosterone synthase deficiency
HPA: Hypothalamo-pituitary-adrenal axis
PE: Physical examination
SD: Standard deviation
NR: Normal range
ACTH: Adrenocorticotropic hormone
PRA: Plasma renin activity
T: Testosterone
P: Progesterone
A4: Androstenedione
17-OHP: 17-hydroxyprogesterone
FC: Fludrocortisone
StAR: Steroidogenic acute regulatory
Na: Natrium / sodium
K: Potassium / kalium
Cl: Chloride

## References

[1] Bockenhauer D, Zieg J (2014). Electrolyte disorders. Clin Perinatol.

[2] Bizzarri C, Pedicelli S, Cappa M, Cianfarani S (2016). Water balance and ‘salt Wasting' in the first year of life: the role of aldosterone-signaling defects. Hormone Res Paediatr.

[3] Root AW. Disorders of aldosterone synthesis, secretion, and cellular function. Curr Opin Pediatr. 2014;26(4):480-6. 10.1097/MOP.0000000000000104. 10.1097/MOP.0000000000000104 24840884

[4] Tseng M-H, Huang J-L, Huang S-M, Tsai J-D, Wu T-W, Fan W-L (2020). Clinical features, genetic background, and outcome in infants with urinary tract infection and type IV renal tubular acidosis. Pediatr Res.

[5] Pai B, Shaw N, Högler W (2012). Salt-losing crisis in infants—not always of adrenal origin. Eur J Pediatr.

[6] Melati W, Ma H, Zhang J, Minlian D, Yanhong L, Qiuli C (2019). Etiology of primary adrenal insufficiency in children: a 29-year single-center experience. J Pediatr Endocrinol Metab.

[7] Perry R, Kecha O, Paquette J, Huot C, Van Vliet G, Deal C (2005). Primary adrenal insufficiency in children: twenty years experience at the Sainte-Justine Hospital, Montreal. J Clin Endocrinol Metab.

[8] Bornstein SR, Allolio B, Arlt W, Barthel A, Don-Wauchope A, Hammer GD (2016). Diagnosis and treatment of primary adrenal insufficiency: an Endocrine Society clinical practice guideline. J Clin Endocrinol Metab.

[9] Speiser PW, Azziz R, Baskin LS, Ghizzoni L, Hensle TW, Merke DP (2010). Congenital adrenal hyperplasia due to steroid 21-hydroxylase deficiency: an Endocrine Society clinical practice guideline. J Clin Endocrinol Metab.

[10] Ferrari P, Bianchetti MG. Diagnostic Investigations in Inherited Endocrine Disorders of Sodium Regulation. 2011:210–34. 10.1159/000327410.

[11] Malikova J, Flück CE (2014). Novel insight into etiology, diagnosis and Management of Primary Adrenal Insufficiency. Hormone R Paediatr.

[12] White PC (2004). Aldosterone synthase deficiency and related disorders. Mol Cell Endocrinol.

[13] Turan I, Kotan LD, Tastan M, Gurbuz F, Topaloglu AK, Yuksel B (2018). Molecular genetic studies in a case series of isolated hypoaldosteronism due to biosynthesis defects or aldosterone resistance. Clin Endocrinol.

[14] Geller DS, Zhang J, Zennaro M-C, Vallo-Boado A, Rodriguez-Soriano J, Furu L (2006). Autosomal dominant Pseudohypoaldosteronism type 1: mechanisms, evidence for neonatal lethality, and phenotypic expression in adults. J Am Soc Nephrol.

[15] Kuribayashi I, Kuge H, Santa RJ, Mutlaq AZ, Yamasaki N, Furuno T (2003). A missense mutation (GGC[<sup>435</sup>Gly]→AGC [Ser]) in exon 8 of the CYP11B2 gene inherited in Japanese patients with congenital Hypoaldosteronism. Hormone Res Paediatr.

[16] Nguyen H-H, Hannemann F, Hartmann MF, Malunowicz EM, Wudy SA, Bernhardt R (2010). Five novel mutations in CYP11B2 gene detected in patients with aldosterone synthase deficiency type I: functional characterization and structural analyses. Mol Genet Metab.

[17] Li N, Li J, Ding Y, Yu T, Shen Y, Fu Q, et al. Novel mutations in the CYP11B2 gene causing aldosterone synthase deficiency. Mol Med Rep. 2016;13. 10.3892/mmr.2016.4906. 10.3892/mmr.2016.4906 26936515

[18] Lages AS, Vale B, Oliveira P, Cardoso R, Dinis I, Carrilho F (2019). Congenital hyperreninemic hypoaldosteronism due to aldosterone synthase deficiency type I in a Portuguese patient - Case report and review of literature. Arch Endocrinol Metab.

[19] Speiser PW, Arlt W, Auchus RJ, Baskin LS, Conway GS, Merke DP (2018). Congenital adrenal hyperplasia due to steroid 21-hydroxylase deficiency: an Endocrine Society clinical practice guideline. J Clin Endocrinol Metab.

[20] Løvås K, McFarlane I, Nguyen H-H, Curran S, Schwabe J, Halsall D (2009). A novel CYP11B2 gene mutation in an Asian family with aldosterone synthase deficiency. J Clin Endocrinol Metab.

[21] Kayes-Wandover KM, Schindler REL, Taylor HC, White PC (2001). Type 1 aldosterone synthase deficiency presenting in a middle-aged Man1. J Clin Endocrinol Metab.

[22] Walker BR, Andrew R, Escoubet B, Zennaro M-C (2014). Activation of the hypothalamic-pituitary-adrenal Axis in adults with mineralocorticoid receptor Haploinsufficiency. J Clin Endocrinol Metab.

